# Health Care Professionals’ Clinical Skills to Address Vaping and e-Cigarette Use by Patients: Needs and Interest Questionnaire Study

**DOI:** 10.2196/32242

**Published:** 2022-04-11

**Authors:** Mary Metcalf, Karen Rossie, Katie Stokes, Bradley Tanner

**Affiliations:** 1 Clinical Tools, Inc Chapel Hill, NC United States

**Keywords:** clinical skills, vaping, e-cigarettes, nicotine, brief interventions, addiction treatment, health care professionals, continuing education

## Abstract

**Background:**

Widespread vaping and e-cigarette use is a relatively new phenomenon. Youth vaping peaked in 2019, with over 25% of high school students currently vaping. e-Cigarettes are used where smoking is not permitted or as an alternative smoking cessation treatment instead of Food and Drug Administration–approved options. Vaping and e-cigarette use has the potential to harm health, including causing adverse respiratory effects and nicotine addiction. Health care professionals need skills training to help their patients with this relatively new and evolving health problem.

**Objective:**

The aim of this study is to understand health care professionals’ training needs in this subject area to determine the focus for web-based continuing education training.

**Methods:**

We reviewed the literature on clinical aspects of vaping and e-cigarette use. Using the results and our experience in substance use continuing education, we created a list of key clinical skills and surveyed health care professionals about their training needs. We also asked about their interest in a list of related topics. We recruited individuals who completed our web-based courses on substance use, members of health care professional–related groups, and experts who had published an article on the subject. Half of the 31 health care professionals who completed the survey were physicians and the remainder were primarily nurses, social workers, and counselors. Participants self-identified as nonexperts (n=25) and experts (n=6) on vaping.

**Results:**

Participants who were nonexperts on average agreed or strongly agreed that they needed training in each of 8 clinical skills (n=25; range 3.7-4.4 agreement out of 5). The top two skills were recommending treatments for patients (4.4 out of 5, SD 0.49) and evaluating and treating the health effects of vaping and e-cigarette use (4.4 out of 5, SD 0.50). Experts agreed on the importance of training for health care professionals in all skills but rated the need for training higher than nonexperts for each topic. Over half of the participating health care professionals (44%-80%) were interested in nearly all (9/10, 90%) vaping-related topics on a checklist. The topics participants were most interested in were the pros and cons of vaping versus smoking and the health effects of second- and third-hand vaping. Primary care physicians showed more interest in vaping-related topics than nonprimary care physicians (*t*_13_=2.17; *P*=.02).

**Conclusions:**

This study confirmed gaps in health care professionals’ vaping-related clinical skills identified in the literature by identifying a perceived need for training in related skills and health care professionals’ interest in key topics related to vaping prevention and cessation. This study provides specific guidance on which clinical skills training is most needed and which topics are most interesting to health care professionals.

## Introduction

### Background

The growth of e-cigarette devices, after their initial promotion as safer alternatives to traditional cigarettes, brought about a disturbing trend of youth vaping, fueled in part by marketing directed to this age group [1] that minimized potential harm [2]. Whereas current cigarette use by high school students steadily decreased in the past 20 years to only 9.4% by 2019 [3], vaping, especially nicotine vaping, was on the rise. By 2019, when we conducted this study, 27.5% of high school students were vaping [4-6]. Vaping by youth later continued at a significant rate of 11% in early 2021 despite less time spent inside schools owing to the pandemic [7]. Many adults who use e-cigarettes as smoking cessation treatment alternatives to Food and Drug Administration (FDA)–approved options end up using both forms of nicotine [8].

Nicotine’s harmful health effects were already known to medical science when nicotine vaping became popular, including nicotine’s addictive properties [9,10] and harmful cardiovascular effects [11-13]. Other harmful health effects became evident over time, including a greater potential for nicotine overdose with vaping than with cigarette smoking, as well as harm to the lungs related to the inhalation of toxic chemicals in vaping liquids [10,11,14,15]. The serious lung disease, e-cigarette or vaping product use–associated lung injury, primarily associated with a vaping liquid additive, underscored the potential for respiratory damage, affecting over 2000 individuals and causing 68 deaths, mainly in the latter half of 2019 [16]. The long-term effects of inhaling various harmful ingredients are not yet fully understood.

With the addictive potential and health effects of vaping and e-cigarette use and increased use by their patients, health care professionals need to know how to prevent and address the use of these products. In the absence of large-scale studies on the best approach to preventing and treating vaping and e-cigarette use, many health care professional societies and addiction specialists recommended following existing evidence-based guidelines for tobacco cessation [17-21]. For example, the questions of the 5 As approach often recommended for tobacco cessation can be modified to pertain to the differences between vaping and e-cigarette use and other tobacco use, such as the nicotine delivery method and typical patterns of use [22]. Professionals also need sufficient knowledge to provide patient and parent education. Tobacco interventions by primary care providers are effective, including for adolescents [23,24]. Educational programs on best practices in this area need to be fluid, as further research gathers data on the best clinical approach to preventing and treating vaping and e-cigarette use by patients. Similarly, the use of e-cigarettes by many people to help with tobacco cessation requires further research [25]. Health care professionals need to understand that the FDA has not approved e-cigarettes as an aid to quit smoking and respond to patients who are using them for this purpose [26].

As a group, the authors brought significant experience in developing nicotine and cigarette addiction cessation training programs [27] and substance abuse cessation training for health care professionals [28-35]. With this experience, we were familiar with the evidence that health care professional screening and interventions effectively improve patient outcomes for substance use problems [36,37], including for tobacco cessation in children and adolescents [38,39]. This experience formed a solid basis to begin developing a curriculum and content.

To address the challenge of determining health care professionals’ greatest needs and interests, we conducted a literature review to identify evidence-based protocols and current thinking on clinical skills thought to be effective for addressing patient vaping and e-cigarette use. We refined the list of skills that we identified with input from expert consultants in vaping and e-cigarette use and using our experience with addiction treatment education. We followed this with a needs analysis using a web-based survey asking health care professionals about the clinical skills training they most needed and topics of most interest to them.

### Objectives

This study aims to determine the need and interest health care professionals have in training on clinical skills to address vaping and e-cigarette use by their patients. We also aim to prioritize the needs and determine differences by groups of clinicians or level of expertise.

## Methods

### Literature Review

The newness of widespread vaping and e-cigarette use and the emerging recognition of the associated adverse health impacts and limits as a tobacco cessation tool created a challenge in developing an evidence-based continuing education program for health care professionals on the topic. To address this challenge, we completed an extensive literature review from 2014 to 2019. Using PubMed, Google Scholar, and PsycINFO, we searched for research-based articles on vaping and e-cigarette use, covering the topics of brief interventions, epidemiology, motivations, prevention, risk factors, health effects, psychosocial impact, addiction, knowledge and practice gaps, clinical guidelines, vaping products and ingredients, and regulations. We used standard internet searches to identify available training for health care professionals on the topic, Centers for Disease Control and Prevention updates, clinical guidelines, and news. Additional keywords used in the internet and web-based searches included *gateway drugs*, *e-cigarettes*, *vaping*, *e-liquid*, *vaporizers*, and variants of these terms. We also searched for previous studies examining health care professional’ perspectives and knowledge on e-cigarette use and vaping and current, evidence-based recommendations for a prevention and treatment clinical protocol. We created survey questions from the clinical skills and related topics most recommended in the literature.

### Ethical Considerations

This research was considered exempt from institutional research board review because it involved survey procedures, and the information obtained could not be linked to the participants and did not place them at risk (approval number: 2019/007).

### Recruitment

Owing to the formative, exploratory nature of this study and a condensed time frame required by the funding mechanism, we used a convenience sample. We recruited and enrolled participants from October 21, 2019, to November 14, 2019, via direct email. Recruitment efforts included contacting the first 100 health care professionals who had taken a Clinical Tools addiction-related continuing education activity a year previously and inviting participants to share the invitation with colleagues. We also contacted approximately 100 health care professionals using social media and email lists. We modified recruitment to achieve participant diversity similar to the distribution in health care professionals. To obtain input from experts in the field, we also emailed 50 authors of research articles on the clinical aspects of vaping and e-cigarettes. We linked to the human participants’ or institutional review board exemption information in the emails. Completing the survey after reviewing that information signified enrollment. Identifying information and study data were stored separately. Participants completing the web-based survey received a US $20 gift card. We checked IP addresses for any duplicate submissions from the same computer.

### Data Collection and Analysis

We developed survey questions on potential clinical training needs and key topics by gathering recommendations from the existing literature on how to address patient vaping [17,18,40] and substance use interventions [41-45], as well as our experience of providing training on addiction for health care professionals for over 20 years. Questions were based on evidence-based clinical skills and topics recommended most frequently; gaps in knowledge, skills, and practice; and general principles of addiction assessment and treatment, particularly for tobacco cessation. Question types included Likert-style questions (5-point agreement) about the perceived need for training in key clinical skills, a checklist of topics related to patient vaping, an open-ended question asking whether they had other related needs and interests, and multiple-choice knowledge questions about common vaping myths. Survey questions included a not applicable or do not know option; all survey questions were required. We administered the needs analysis web-based survey from October 21, 2019, to November 16, 2019.

We asked about the need for clinical skills training, interest in vaping topics, and knowledge of several common myths about vaping. We also asked participants to identify their level of expertise in the field of vaping and e-cigarettes (expert or nonexpert) and whether or not they worked in primary care. We asked both experts and nonexperts about the training needs of nonexperts by changing the question stem for each group.

We calculated average Likert rating scores and their SDs for the skills and knowledge data, plus the percentage of participants rating each item with *agree* or *strongly agree*. For the topic interest data, we calculated the percentage of participants who endorsed each topic. We calculated *t* scores and *P* values to compare the overall results in the groups (expert vs nonexpert and primary care physicians vs not primary care physicians). Descriptive statistics were used to analyze and compare the results for the individual questions across groups. A single response to the optional open-ended question is reported without analysis.

## Results

### Literature Review

The literature review identified several gaps in health care professionals’ medical knowledge, clinical skills, and practice related to vaping and e-cigarettes [46-50]. The gaps in health care professionals’ knowledge were of health effects [48,49], vaping methods, products, and patterns of use; recommended interventions; and the effectiveness and safety of e-cigarettes to aid smoking cessation. Gaps in clinical skills or practice were evident in health care professionals’ infrequent practice of screening [51], following up on screening results [52], and providing interventions [48,49]. Health care professionals screen patients for the use of noncigarette products and advise quitting them less frequently than they do for cigarettes [46]. Health care professionals also need to be prepared to provide patient education and recommend resources, such as social media or SMS text messaging support for vaping or e-cigarette cessation [53].

The clinical guidance from national professional organizations available at the time of this search in September 2019 was limited and included guidelines by the American Academy of Pediatrics recommending tobacco cessation counseling and FDA-approved tobacco dependence pharmacotherapies [20] and clinical tips sheets by the American Academy of Family Physicians [54]. Another resource was the Surgeon General’s report on e-cigarettes and young people [10]. Several journal articles drew conclusions about effective prevention interventions, identified youths and young adults at greater risk, and recommended counseling interventions based on the available evidence [40,50,55-57]. The search also revealed only a few, often expired, web-based trainings for health care professionals on the topic [40,58-62]. Boston Medical Center and Montreal University subsequently published a guideline based on tobacco cessation interventions and expert opinion [18]. As of this study, there were no nationally recognized clinical guidelines for vaping cessation treatment (2020). However, a curriculum was developed subsequently for pediatricians by the American Academy of Pediatrics in 2021 [63].

We consolidated the literature review results to produce a list of skills and topics generally considered by experts in the field as important for clinicians to understand. This list is reflected in the survey questions.

### Participants

A total of 31 health care professionals participated in the needs analysis surveys and self-identified as nonexperts (25/31, 81%) or experts (6/31, 19%) in vaping. Nonexperts included physicians (15/25, 60%), nurses (4/25, 16%), social workers or counselors (3/25, 12%), and other health care professionals (3/25, 12%). Self-identified experts on vaping were physicians (3/6, 50%), a psychologist (1/6, 17%), an epidemiologist (1/6, 17%), and a social worker (1/6, 17%). Physicians were divided nearly evenly between primary care (8/15, 53%) and nonprimary care (7/15, 47%).

The diversity of the sample reflected ethnic and racial percentages of the professions in US physicians and nurses [64,65], except for low representation by Black participants. The demographics, voluntarily described by participants, were Asian 19% (6/31), Black 0% (0/31), White 68% (21/31), >1 race 10% (3/31); other 3% (1/31), and Hispanic or Latino 3% (1/31; prefer not to answer 3/31, 9%). More participants were women (22/31, 71%) than were men (9/31, 29%).

### Survey Results and Analysis

#### Clinical Skills Training Needs

For clinical skills training, we asked participants to rate their perceived need for training in 8 clinical skills, listed in Table 1, using a Likert-type survey with a 5-point agreement scale.

**Table 1:** Health care professionals’ perceived need for clinical skills training on vaping (N=31).

“I or Healthcare Professionals NEED MORE TRAINING in how to”	Nonexpert (n=25)		Expert (n=6)	
	Rating^a^, average (SD)	Agree or strongly agree, n (%)	Rating^a^, average (SD)	Agree or strongly agree, n (%)
Recommend treatments for patients who vape or use electronic cigarettes (e-cigarettes)	4.4 (0.49)	25 (100)	4.8 (0.41)	6 (100)
Evaluate and treat health effects in patients who vape or use e-cigarettes	4.4 (0.50)	25 (100)	4.7 (0.52)	6 (100)
Provide brief interventions for patients who vape tetrahydrocannabinol	4.3 (0.69)	22 (88)	4.8 (0.41)	6 (100)
Helping patients who are using e-cigarettes to quit smoking	4.1 (0.78)	21 (84)	4.5 (0.82)	5 (83)
Talk with parents about vaping prevention or helping their adolescent child quit	4.1 (0.85)	20 (80)	5.0 (0)	6 (100)
Counsel patients about how to quit vaping or e-cigarette use	4.0 (0.75)	21 (84)	4.8 (0.52)	6 (100)
Motivate patients to quit vaping or e-cigarette use	4.0 (0.79)	20 (80)	4.8 (0.52)	6 (100)
Assess vaping and e-cigarette use in patients	3.7 (0.95)	20 (80)	4.3 (0.82)	6 (100)

^a^Likert rating: 1=strongly disagree, 2=disagree, 3=neither agree nor disagree, 4=agree, 5=strongly agree.

Participants generally agreed that they need training in clinical skills to address their patients’ vaping or e-cigarette use. The ratings by all participants are presented first, followed by comparisons of expert opinion versus nonexpert opinion and primary care physicians versus nonprimary care physicians.

A majority of participants agreed that clinicians need more training for all 8 clinical skills listed (Table 1). The skills that health care professionals who were not experts on vaping (n=25) most often agreed that they required training were as follows: (1) recommend treatments for patients who vape or use e-cigarettes (average 4.4, SD 0.49; 25/25, 100% agree or strongly agree) and (2) evaluate and treat health effects in patients who vape or use e-cigarettes (average 4.4, SD 0.5; 25/25, 100% agree or strongly agree).

Nonexperts (n=25) and experts (n=6) generally agreed about the need for training in the same clinical skills. However, nonexperts’ average ratings ranged lower than experts’ ratings (3.7 to 4.4 out of 5 vs 4.3 to 4.8 out of 5).

Experts agreed that all skills listed were needed, rating none of the skills below 4 (agreement). According to experts, the skills needed the most were as follows: (1) talk with parents about vaping prevention or helping their adolescent child quit (average 5.0, SD 0; 25/25, 100% agree or strongly agree) and (2) four other skills were rated nearly as highly (average rating 4.8, SDs 0.41-0.52; 25/25, 100% agree or strongly agree). These pertained to patient counseling, motivating patients to quit, recommending treatment, and providing brief interventions for patients who vape tetrahydrocannabinol (THC).

The clinical skills with the largest rating differences between the expert and nonexpert groups (0.8-0.9 points) were talking with parents about vaping prevention or helping their adolescent child quit, counseling patients about how to quit vaping or e-cigarette use, and motivating patients to quit vaping or e-cigarette use.

The clinical skills that primary care physicians (n=8) on average agreed most strongly that they need training in were as follows: (1) evaluate and treat health effects in patients who vape or use e-cigarettes (average rating 4.8 out of 5, SD 0.46; 8/8, 100% agree or strongly agree), (2) recommend treatments for patients who vape or use e-cigarettes (average rating 4.6 out of 5, SD 0.51; 8/8, 100% agree or strongly agree), (3) provide brief interventions for patients who vape THC (average rating 4.6 out of 5, SD 0.52; 7/8, 88% agree or strongly agree), and (4) counsel patients about how to quit vaping or e-cigarette use (average rating 4.4 out of 5, SD 0.74; 7/8, 88% agree or strongly agree).

Primary care physicians on average agreed more strongly that they needed training in the 8 clinical skills listed than did nonprimary care physicians, averaging 4.3 out of 5 (SD 0.34) versus 3.9 out of 5 (SD 0.37; t_13_=2.56; 95% CI for the difference 0.0734-0.8266; *P*=.01).

#### Topics of Interest

We asked nonexperts in vaping (n=25) to indicate which topics relevant to vaping interested them on a checklist of 10 topics, listed in Table 2. Most of the nonexpert participants indicated an interest in most (9/10, 90%) of the topics listed, with the percentage of participants interested in each topic ranging from 52% to 80% (Table 2). Topics endorsed most often were *Pros and cons of vaping versus smoking*, selected by 80% (20/25) and *Health effects from second- and third-hand vaping*, selected by 76% (19/25).

**Table 2:** Number of health care professionals who are not experts on vaping who indicated an interest in vaping-related topics (n=25).

Topics related to vaping and e-cigarettes (in order from most often selected to least)	Health care professionals endorsing interest in topic, n (%)
Pros and cons of vaping vs smoking	20 (80)
Health effects from second- and third-hand vaping	19 (76)
Risks of vaping specifically	18 (72)
Vaping and e-cigarette devices, liquids, and their ingredients	17 (68)
Vaping prevention	16 (64)
Biology of endocannabinoids and pharmacology of tetrahydrocannabinol	16 (64)
Special needs regarding vaping because of cultural, racial, ethnic, or socioeconomic differences	14 (44)
Pathology and radiology of e-cigarette and vaping-associated lung illness	13 (52)
Patient and parent resources on these topics	13 (52)
Biology of the nicotine system and pharmacology of nicotine	13 (52)
None of the above	1 (4)

Physicians (n=15), all of whom were not experts in vaping, were most often interested in *Health effects from second- and third-hand vaping* (n=13, 87%) and *Pros and cons of vaping versus smoking* (n=12, 80%). The fewest physicians were interested in the topics *Special needs regarding vaping due to cultural, racial, ethnic, or socioeconomic differences* (5/15, 33%) and *Biology of the nicotine system and pharmacology of nicotine* (5/15, 33%).

Primary care physicians (n=8) on average showed interest in significantly more topics than nonprimary care physicians (n=7): 5.5 topics (55%, SD 1.51%) versus 3.6 topics (36%, SD 1.4%; t_13_=2.168; *P*=.02).

We also offered participants the opportunity to add skills or interests that were not included via an open-ended question. In response to this optional, open-ended question, a hospital staff participant identified “negative consequences of vaping.” No other participants responded to this question.

#### Vaping Knowledge

We asked participants to indicate their level of agreement with 3 myths about vaping, described in Table 3, using the same Likert-type survey style.

**Table 3:** Health professionals’ agreement with myths about vaping.

Question: rate your agreement or disagreement with the following statements about vaping	Nonexpert (n=25)			Expert (n=4)	
	Rating^a^, average (SD)	Disagree or strongly disagree, n (%)	Rating, average (SD)		Disagree or strongly disagree, n (%)
Vaping or electronic cigarette use is a good option for smokers trying to quit (correct answer is 1-2, strongly disagree or disagree).	1.8 (0.94)	14 (56)	1.8 (0.96)		3 (75)
Vaping tetrahydrocannabinol is the main problem. Vaping of nicotine and flavored liquids is not significant clinically (correct answer is 1-2, strongly disagree or disagree).	1.8 (0.75)	22 (88)	1.5 (0.58)		4 (100)
Helping patients who vape is just like helping smokers or drug users (correct answer is 1-2, strongly disagree or disagree).	3.5 (0.85)	3 (16)	2.2 (1.26)		3 (75)

^a^Likert rating: 1=strongly disagree, 2=disagree, 3=neither agree nor disagree, 4=agree, 5=strongly agree.

In response to 3 Likert-type agreement–style knowledge questions, both vaping expert and nonexpert participants were fairly knowledgeable about several common myths about vaping. Their responses aligned with the correct answer, which was to disagree with the inaccurate statements (Table 3). Experts disagreed more strongly with the statements. Differences were not statistically significant on average. However, 1 statement, “Helping patients who vape is just like helping smokers or drug users,” had a relatively larger difference (1.3 out of 5) between nonexperts and experts, suggesting a larger gap in the knowledge about this topic.

Participants were also asked, “Compared to quitting cigarettes, quitting vaping of nicotine is (easier, same, harder).” Most participants (24/31, 77%) said it was the same; 19% (6/31) said it was harder, and 6% (2/31) said it was easier to quit vaping. The ratings for experts and nonexperts were similar.

## Discussion

### Principal Findings

To confirm and prioritize health care professional training gaps in clinical skills and knowledge related to vaping and e-cigarette use, we conducted a needs analysis with health care professionals and experts in vaping. We asked about their need for training in clinical skills and their interest in related topics. To explore whether there was a knowledge gap, we also asked whether they agree with common myths regarding vaping.

Most health care professionals participating in this study agreed that they needed training in key clinical skills on helping patients with vaping, as identified via a literature review. A majority were also interested in learning about a list of key topics related to vaping culled from the literature review. Participant responses to myths about vaping not only showed some awareness of their inaccuracy but also identified areas of misunderstanding. Together, these results suggest that health care professionals perceive a need for training in vaping-related clinical skills, are interested in learning about related key topics, and have a gap in some related knowledge. Results from experts confirmed the need for the clinical skills training and education in key related topics.

The clinical skills that participating health care providers agreed they need involve providing brief interventions, including counseling and motivating on quitting and prevention, recommending treatments, and evaluating and treating the health effects. The finding that training is needed across the range of skills identified in this study highlights the broad training needs of health care professionals in the evolving patient use of vaping and e-cigarettes and associated health concerns.

Experts more strongly agreed that health care professionals need training in each of the clinical skills needed to help their patients with vaping when compared with health care professionals who were not experts on vaping. The reasons for this difference might include health care professionals not being as aware of a training need, providers being better prepared than experts realize, or other reasons, which would require further research to understand.

A majority of participating health professionals who were not experts on vaping were interested in nearly all the vaping-related topics listed in this study. This indicates widespread agreement on what general areas to cover in a vaping training program. The topics with the highest rate of interest, *Pros and cons of vaping versus smoking* (20/25, 80%), *Health effects of second- and third-hand vaping* (19/25, 76%), and *Risks of vaping* (18/25, 72%) can be emphasized.

Further research could explore the reasons for high and low ratings for need and interest to distinguish between lack of interest, lack of relevance for their practice, or already having a particular skill or knowledge.

Participants’ relatively higher agreement with a myth that there are no differences between interventions for vaping and other substance use points to an understanding of the similarities but a knowledge gap about the differences that do exist. This confirms knowledge and skills gaps identified in the literature review. Nonexperts in vaping need a better understanding that there are some differences in clinical treatment for vaping and e-cigarette cessation versus smoking cessation to respond to the reinforcing effects of flavoring, different patterns of use, peer influence, and the ability to vape or use e-cigarettes discretely and in more environments [9,10,17,66,67].

The topics physicians were most interested in differed somewhat from those of participating health care professionals as a whole. They showed greater interest in the health effects of vaping and e-cigarettes, recommending treatments, and providing brief interventions for vaping THC. As might be expected from their patient population, primary care physicians rated the need for training higher than nonprimary physicians (average 4.3, SD 0.34 vs 3.9, SD 0.37, out of 5; *P*=.01). They also indicated more interest in vaping-related topics, endorsing an average of 5.5 versus 3.6 topics (*P*=.02).

The results support a training for health care professionals on vaping and e-cigarettes that emphasizes the primary care setting and the identified skills and topics. However, fairly low SDs throughout the study, despite over half of the participants being nonphysicians and nonprimary care, support the development of a universally applicable training. The training activities must be flexible enough to address the minor differences in needs based on the health care professionals’ work setting and patient population.

### Applications

Subsequent to this research, a few more clinical practice guidelines on vaping and e-cigarettes emerged. For example, in 2021, the American Academy of Pediatrics published an e-cigarette curriculum and poster for pediatricians only accessible to members [63]. As adolescents and young adults use these products more than other age groups, and as that young demographic is less likely to see the physician than older individuals, nonphysician health care professionals need this training in addition to pediatric physicians.

Although our work was specific to vaping cessation, some takeaways can be applied to continuing education development for health care professionals in general. Programs on substance use should include content related to health impacts to increase physician engagement and interest. In addition, content related to clinical skills should focus on specific areas where this research demonstrated gaps between the health care professionals’ self-perceived understanding of their training needs and real-world outcomes, such as the need to adapt counseling and interviewing techniques to the specific substance and cultural, racial, ethnic, and socioeconomic differences. In addition, health care professionals may need to be persuaded that topics favored more highly by experts on a topic are relevant to their practice and patient population.

After identifying health care professionals’ training needs and practice gaps, we developed 3 case-based web-based training activities focused on the clinical skills identified. These activities are currently available in the studies by Rossie [68-70]. In an early evaluation of that program with 78 health care professionals, most users indicated improvement in their knowledge (74/78, 95%) and competence (66/78, 85%). The learners gave high ratings for their vaping-related self-efficacy (4.3 out of 5), intended behavior (4.3 out of 5), and attitude (4.3 out of 5) following the completion of the program. An analysis of the educational impact of the activities is ongoing.

e-Cigarettes and vaping are not the *safe alternative* to cigarette use, as initially presented by the industry. Patients who vape, use e-cigarettes, or are considering using them will benefit from health care professionals who are able and ready to intervene with prevention, evaluation, and cessation strategies. Health care professionals who are adequately trained regarding patient vaping and e-cigarette use will decrease the burden of nicotine, THC, and harmful chemicals used in these products on patient health.

### Key Findings

Most participants agreed that clinicians need more training on vaping across a list of clinical skills commonly used to address substance use. However, experts’ agreement was stronger.

Health care professionals without expertise in vaping understood their need for training in clinical skills related to vaping, as was evident by selecting similar needs to what experts selected. However, expert agreement on needs was significantly stronger, on average.

Health care professionals agreed most strongly that they need clinical skills training in recommending treatments for patients who vape or use e-cigarettes and evaluating and treating health effects in patients who vape or use e-cigarettes.

There was strong interest by most participants who were not experts in vaping in learning about most topics on a list of key topics about vaping. The strongest interest was in the pros and cons of vaping versus smoking and the health effects of second- and third-hand vaping.

Physicians in primary care more frequently expressed interest in topics related to vaping and e-cigarette use than those not in primary care. Physicians had greater interest in the health impact of vaping than other health care professionals.

Participants showed a moderately good understanding of myths about vaping from a clinical perspective but need better awareness of the differences between treating a patient for vaping and other substance use.

### Limitations

This research was limited by searching only 3 major databases of journals in our literature review, PubMed, Google Scholar, and PsycIINFO. For example, we did not search for Scopus and CINAHL, which have relevancy because of the clinical nature of this topic. We may have missed some topics mentioned in articles indexed only in the sources not used.

Limitations related to sampling may have affected the results. We elected to use a convenience sample owing to the time frame and funding available. Thus, the participants may not represent the health care professionals as a whole.

Several limitations are related to study participants. We directed half of our recruitment efforts to a list of health care professionals who had taken our other substance use courses because we wanted to understand the needs of our typical audience. Consequently, participants may have more interest in and knowledge about substance use than the average health care professional. An honorarium provided to participants may have introduced a bias toward participation or more positive feedback. The small number of experts in this study limits comparisons of experts with nonexperts. Although we had participant recruitment efforts that specifically sought to increase the diversity of the sample, African American health care professionals were not represented.

Finally, questions were not randomized owing to limitations of the software used, which may have resulted in items near the top of the checklist being chosen more often than they would have otherwise.

### Conclusions

Using a survey derived from a literature review and expert knowledge, we identified health care professionals’ perception of their need for training in clinical skills, their interests, and their knowledge related to vaping and e-cigarette use by patients. The list of clinical skills training needed and topics of interest for health care professionals identified in this study confirms the need identified in the literature and adds prioritization according to which skills and topics had the greatest need and interest. This information can contribute to the effective focus of the growing body of training on helping patients who vape or use e-cigarettes or considering use. Combining literature review with expert opinion and health care professionals’ interest enabled us to develop a targeted curriculum to address the clinical skills gaps and the evolving health concerns of vaping and e-cigarette use. The results suggest that vaping and e-cigarette use is an area where health care professional training would benefit.

## Abbreviations

FDA: Food and Drug Administration
THC: tetrahydrocannabinol
